# Biofilm Formation of *Helicobacter pylori* in Both Static and Microfluidic Conditions Is Associated With Resistance to Clarithromycin

**DOI:** 10.3389/fcimb.2022.868905

**Published:** 2022-03-25

**Authors:** Paweł Krzyżek, Paweł Migdał, Rossella Grande, Grażyna Gościniak

**Affiliations:** ^1^ Department of Microbiology, Faculty of Medicine, Wroclaw Medical University, Wroclaw, Poland; ^2^ Department of Environment, Hygiene and Animal Welfare, Wroclaw University of Environmental and Life Sciences, Wroclaw, Poland; ^3^ Department of Pharmacy, University “G. d’Annunzio” of Chieti-Pescara, Chieti, Italy

**Keywords:** *Helicobacter pylori*, antibiotic resistance, multidrug resistance, biofilm formation, biofilm matrix, flow conditions, microfluidic system, Bioflux

## Abstract

It is widely accepted that production of biofilm is a protective mechanism against various type of stressors, including exposure to antibiotics. However, the impact of this structure on the spread of antibiotic resistance in *Helicobacter pylori* is still poorly understood. Therefore, the aim of the current research was to determine the relationship between biofilm formation and antibiotic resistance of *H. pylori*. The study was carried out on 24 clinical strains with different resistance profiles (antibiotic-sensitive, mono-resistant, double-resistant and multidrug-resistant) against clarithromycin (CLR), metronidazole (MTZ) and levofloxacin (LEV). Using static conditions and a crystal violet staining method, a strong correlation was observed between biofilm formation and resistance to CLR but not MTZ or LEV. Based on the obtained results, three the strongest and three the weakest biofilm producers were selected and directed for a set of microfluidic experiments performed in the Bioflux system combined with fluorescence microscopy. Under continuous flow conditions, it was observed that strong biofilm producers formed twice as much of biofilm and created significantly more eDNA and in particular proteins within the biofilm matrix when compared to weak biofilm producers. Additionally, it was noticed that strong biofilm producers had higher tendency for autoaggregation and presented morphostructural differences (a greater cellular packing, shorter cells and a higher amount of both OMVs and flagella) in relation to weak biofilm counterparts. In conclusion, resistance to CLR in clinical *H. pylori* strains was associated with a broad array of phenotypical features translating to the ability of strong biofilm formation.

## 1 Introduction

Over 40 years have passed since the Gram-negative bacterium *Helicobacter pylori* was recognized as a leading etiological agent of gastrointestinal pathologies in humans (Denic et al., 2020). Infections most often occur during childhood and lead to the development of a variety of stomach diseases, including chronic gastritis, peptic ulcers or gastric cancers (Schulz and Kupčinskas, 2020). Despite decades of research on the pathophysiology of *H. pylori*, infections caused by this pathogen remain among the most common worldwide (Hooi et al., 2017). Nowadays, the prevalence of primary resistance of *H. pylori* to three most important antibiotics used classically in therapies, i.e., clarithromycin (CLR), metronidazole (MTZ) and levofloxacin (LEV), has exceeded the critical threshold of 15% in virtually all areas monitored by the World Health Organization (WHO) (Savoldi et al., 2018). In addition, the presence of multidrug-resistant isolates of this bacterium, showing insensitivity to all three above-mentioned antibiotics simultaneously, is observed with an increasing frequency (Boyanova et al., 2019). It seems that the easiness to acquire mutations in drug target sites and the need for use of multi-component treatments are strongly related to this dramatic situation (Graham, 2020). Currently, it is becoming widely accepted that antibiotic resistance of *H. pylori* may also be associated with more complex physiological processes such as biofilm formation (Krzyżek et al., 2020; Tshibangu-Kabamba and Yamaoka, 2021).

Biofilm is a highly-structured and spatially arranged conglomerate of microbial cells immersed in the self-produced extracellular matrix, creating an interphase with the surrounding environment (Guzmán-Soto et al., 2021). This structure enables aggregation of microbial producers and enhances their survival in unfavorable conditions (Bowler et al., 2020). Microbial cells in the biofilm state are characterized by an altered gene expression profile, but also possess a different rate of multiplication, metabolism and morphology compared to planktonic forms (Penesyan et al., 2021). Biofilms are often found in nature, hence this term has historically been used extensively in the environmental microbiology (Flemming et al., 2021). Nowadays, however, more attention is paid to their presence in the human body, where they can affect both the health maintenance or the development of diseases (Jamal et al., 2018). According to the US National Institutes of Health and the US Centers for Disease Control and Prevention, biofilms are responsible for over 80% of all human infections and 60% of those of a chronic nature (Rumbaugh and Sauer, 2020). One of the most important features of biofilm is its drastically reduced sensitivity to antibiotics and other antimicrobial compounds (up to 1000 times) compared to planktonic forms (Hall and Mah, 2017; Khan et al., 2021; Singh et al., 2022). A number of features are responsible for this phenotype, including a slower growth rate and presence of persister cells, increased activity of efflux pumps (transporters removing antibiotics from the cellular interior), as well as a thick matrix layer limiting the diffusion of antimicrobial compounds in the local environment. There is plenty of scientific evidence showing the relationship not only between the composition of the biofilm matrix and antibiotic resistance (Pozzi et al., 2012; Billings et al., 2013; McCarthy et al., 2015; Mongkolrob et al., 2015; Dominguez et al., 2019), but also between the intensity of production/thickness of this biostructure and the prevalence of resistance to selected groups of antibiotics (Neupane et al., 2016; Belbase et al., 2017; Tahaei et al., 2021; Saber et al., 2022) or even multidrug resistance (Bardbari et al., 2017; Belbase et al., 2017; El-Zamkan and Mohamed, 2021; Ratajczak et al., 2021; Sherif et al., 2021). Hence, many scientists indicate that the design of an appropriate antimicrobial therapy depends largely on an understanding of mechanisms determining maturation and establishment of biofilms (Boudarel et al., 2018; Bowler et al., 2020).

Knowledge about the physiology and processes governing biofilm formation of *H. pylori* is still superficial (Krzyżek et al., 2020). This situation has resulted from the prolonged refusal of the scientific society to accept both the existence of biofilms in the human digestive system (Macfarlane and Dillon, 2007; Motta et al., 2021) and the ability of *H. pylori* to form biofilm in the stomach (Carron et al., 2006). Fortunately, in the last five years this impasse has been gradually broken, contributing to the release of pioneering research characterizing biofilm forms of this bacterium (Krzyżek et al., 2020). Original papers focusing on this subject showed that biofilm *H. pylori* forms exhibit significantly higher levels of tolerance to various classes of clinically used antibiotics (Attaran et al., 2017; Yonezawa et al., 2019; Cai et al., 2020; Fauzia et al., 2020; Hathroubi et al., 2020b). Furthermore, reports identifying components of the biofilm matrix produced by *H. pylori* indicated that these consist mainly of proteins (Hathroubi et al., 2018b; Windham et al., 2018; Hathroubi et al., 2020a; Hathroubi et al., 2020b) and extracellular DNA (eDNA) (Grande et al., 2015; Hathroubi et al., 2018b; Windham et al., 2018), and may in part contribute to the above-mentioned reduction of antibiotic sensitivity (Hathroubi et al., 2020b). These results confirmed the earlier hypothesis about the significant influence of biofilm on the eradication of *H. pylori* and became an incentive for the scientific community to expand knowledge on this important subject (Hathroubi et al., 2018a; Rizzato et al., 2019; Krzyżek et al., 2020).

In response to the above-presented urgency, the aim of this original article was to determine the relationship between antibiotic resistance and ability to form biofilm by clinical *H. pylori* strains. Furthermore, for the first time for this bacterium, a comparative analysis of biofilms created by different *H. pylori* strains was performed under microfluidic conditions.

## 2 Materials and Methods

### 2.1 Bacterial Strains

A total of 24 *H. pylori* strains isolated from primary infected (non-eradicated) patients with gastritis were used in this study. These strains were isolated between 2015-2020 and now belong to the collection of the Department of Microbiology, Wrocław Medical University in Poland. The selection of strains was dictated by their resistance profile and included three representatives of each of the following groups: antibiotic-sensitive, mono-resistant (CLR, MTZ or LEV), double-resistant (CLR+MTZ, CLR+LEV and MTZ+LEV) and multidrug-resistant (CLR+MTZ+LEV) (**Supplementary Table 1**). The determination of resistance of *H. pylori* strains was routinely performed immediately after their isolation using E-tests (bioMérieux, Marcy l’Etoile, France). According to the EUCAST recommendations the resistance breakpoints were > 0.5 µg/mL, > 8 µg/mL and > 1 µg/mL for CLR, MTZ and LEV, respectively (EUCAST, 2022). Presence of resistance to two other routinely determined antibiotics, amoxicillin (AMX, > 0.125 µg/mL) and tetracycline (TET, > 1 µg/mL), has been excluded. Two *H. pylori* reference strains (antibiotic sensitive - ATCC 51932 and mono-resistant to CLR – ATCC 700684, both obtained from the American Type Culture Collection) were additionally used for comparison in experiments determining biofilm formation with a crystal violet staining method. All tested strains were stored in glycerol stocks at -80°C and revitalized by subculturing twice on Columbia agars (Difco, Lublin, Poland) with 10% horse blood and incubating each time for three days at 37°C and microaerophilic conditions (Genbox microaer kits, BioMerieux, Marcy I’Etoile, France).

### 2.2 Determination of Biofilm Formed by *H. pylori* Strains

#### 2.2.1 Static Conditions

Bacterial suspensions with a density of approx. 10^8^ CFU/mL (4 McFarland units) were prepared in Brain Heart Infusion (BHI; Oxoid, Dardilly, France) broth with 2% fetal calf serum (FCS; Gibco, Paisley, Scotland, UK). A 1 mL of the obtained suspensions was added to each well of a flat-bottom, ventilated 12-well microtiter plate (Bionovo, Legnica, Poland) and subjected for three days of incubation at 37°C, microaerophilic conditions and gentle shaking of 50 rpm (MaxQ 6000, Thermo Fisher, Waltham, MA, USA) (Hathroubi et al., 2018b). The measurement of biofilm amount using a crystal violet staining method was performed on the basis of Hathroubi et al. (2018b) with a slight modification including the fixation of biofilm samples at 60°C instead of the air-drying (Reuter et al., 2010; Zhang et al., 2017). After the incubation period, the entire bacterial suspension was collected from the microtiter plates, the wells were rinsed with 1 mL of PBS (Sigma-Aldrich, St. Louis, MO, USA) and then filled with 1 mL of 96% ethanol (Stanlab, Lublin, Poland). After 5 min, the ethanol was removed, the wells were rinsed again with 1 mL of PBS, and the plates were dried at 60°C for 15 min to attach the remaining biofilm to the plates’ walls. After this, 2 mL of 0.1% crystal violet solution (Sigma-Aldrich, St. Louis, MO, USA) was added to each well and such plates were submitted for a 15-min incubation. After this step, the dye was removed, the plates were washed again with 1 mL of PBS, and then 1 mL of 96% ethanol was added to the empty wells to dissolve the biofilm-absorbed crystal violet. A 0.2 mL of the solutions was withdrawn from each well and transferred to a 96-well microtiter plate (Bionovo, Legnica, Poland). The absorbance was measured at 590 nm (OD_590_) with an Asys UVM 340 microplate reader (Biochrom Ltd., Cambridge, UK). The tests were performed in three biological replications with six technical repetitions (n ​​= 18/strain).

On the basis of the obtained results, the strains were categorized in terms of their ability to create biofilms using the classification of Fauzia et al. (2020), with a slight modification including the addition of medium producers of these structures. The *H. pylori* strains were classified as strong, medium and weak biofilm producers when the OD_590_ of the crystal violet solution was equal to ≥ 0.4, 0.4 - 0.3, 0.3 - 0.2, respectively. Values obtained for the pure medium, being a negative control, were ​​< 0.2 and indicated the lack of biofilm production by the tested strain.

#### 2.2.2 Flow Conditions

The biofilm formation under flow conditions was determined using 48-well microfluidic plates and the Bioflux 1000 system (both from Fluxion, San Francisco, CA, USA). In the validated version of the experiment, 0.1 mL of BHI broth + 2% FCS was added to inlet wells of the microfluidic plate. Microcapillaries were rinsed with a strong stream of the medium (10 dyne/cm^2^) for 10 sec, thus unblocking the canals, and then the medium was left for 15 min to unable the components to precoat the walls of microcapillaries. Next, the inlet wells were emptied and 1 mL of a bacterial suspension (10^8^ CFU/mL) in BHI broth + 2% FCS was added to each of them. After this step, the medium flow was switched from the inlet to the outlet wells at a rate of 0.1 dyne/cm^2^ for 24 h (during the validation process, the speed of 0.15 dyne/cm^2^ and 0.2 dyne/cm^2^ were also determined, and after selecting 0.1 dyne/cm^2^ also longer incubation periods of 48 h and 72 h were verified). This stage of the experiment was carried out at 37°C and microaerophilic conditions (Pecon Incubator XL S1, Carl Zeiss, Jena, Germany). After the designated incubation period the flow of the medium was stopped, the inlet wells were emptied from the remaining medium and then filled with a solution containing three fluorescent dyes: 0.3 µL of SYTO9 (L10316, ThermoFisher, Waltham, MA, USA), 1 µL of DAPI (62248, ThermoFisher, Waltham, MA, USA) and 100 µL of SYPRO RUBY (F10318, ThermoFisher, Waltham, MA, USA), enabling for determination of the bacterial biomass, extracellular DNA (eDNA) and extracellular proteins of the biofilm matrix, respectively (Cheng et al., 2021). The medium flow was switched from the inlet to the outlet wells at a rate of 0.1 dyne/cm^2^ and a period of 1 h. Photographs from the experiments were taken using an inverted fluorescence microscope (GmbH, Jena, Germany) and were analyzed using integrated with this system the Bioflux Montage software (Fluxion, San Francisco, CA, USA). The tests were performed in six biological replications with three technical repetitions constituting three different fragments of microcapillaries (n = 18/strain).

With the help of the Bioflux Montage software specific parameters were calculated from the obtained photographs, i.e., the degree of microcapillary coverage (being a measure of the biofilm formation), the fluorescence intensity and the degree of co-localization of fluorescence signals (eDNA/cells and proteins/cells co-localizations, indicating the rate of bacterial coverage by the matrix components), and the amount of bacterial aggregates with given dimensions (information about the tendency for autoaggregation). In the first stage, the size of aggregates was normalized to dimensions of the microcapillary canal in the examined photo and clusters of bacterial cells corresponding to > 1%, 1% - 0.5%, 0.5% - 0.1% and < 0.1% of the microcapillary size were categorized as large, big, medium and small, respectively. Results with a value less than 0.0004%, representing the size of a coccoid form of *H. pylori*, were considered as artefacts and thus were rejected. In the second stage, the total area occupied by aggregates of a give category was calculated and presented as a percentage share in all aggregates formed. The analysis was performed from all six biological replications of experiments carried out in microfluidic conditions (n ​​= 6/strain).

In a primary version of the study, an attempt was also made to determine the capacity of *H. pylori* to form biofilms from the preformed microcolonies subjected to the flow of fresh medium without bacterial cells (this model was rejected during the validation stage, see below). The first stage of flushing the microcapillaries and precoating them with the components of medium was similar to the version of the experiment described above. After this step, 0.1 mL of BHI broth + 2% FCS with a bacterial suspension (10^8^ CFU/mL) was added to the outlet wells. The suspension was flown towards the outlet to the inlet wells at a rate of 5 dyne/cm^2^ for 5 sec and then submitted for a 1-h adhesion of bacteria. After this period, 0.9 mL of BHI + 2% FCS was added to the inlet wells and the flow towards the inlet to the outlet wells was started with a speed of 0.1 dyne/cm^2^ for 24 h at 37°C and microaerophilic conditions. As for all the six tested *H. pylori* strains the dimensions of primary adhered bacterial cells/aggregates were identical to those obtained at the end of the experiments, their analysis was not performed and the model was finally abandoned.

### 2.3 The Real-Time Determination of the Autoaggregation Degree

The real-time autoaggregation measurement was performed using an inverted fluorescence microscope (GmbH, Jena, Germany) and the Bioflux Montage software (Fluxion, San Francisco, CA, USA). For this purpose, 1 mL of BHI broth + 2% FCS with a bacterial suspension (10^8^ CFU/mL) was added to the inlet and outlet wells of the 48-well microfluidic plates (Fluxion, San Francisco, CA, USA), however in this case the medium flow was not switched on. For the better visualization of bacterial cells 1 µL of the non-toxic dye from the CellTrace CFSE Cell Proliferation Kit (C34554, ThermoFisher, Waltham, MA, USA) was also added to the medium. Before starting the experiment, the bacteria were incubated with the dye-containing medium for about 10 min at 37°C. Then, pictures of bacteria incubated at 37°C and microaerophilic conditions were taken every minute for a period of 1 h. After the completion of experiments the rate of coverage of the observation field was calculated from the obtained photos and interpreted as the degree of autoaggregation. The autoaggregation at a given time point (T_1_ – T_60_) was computed by subtracting the initial autoaggregation value in the sample (T_0_). The tests was performed in three biological replications (n ​​= 3/strain).

### 2.4 Determination of the Morphostructure of Aggregates and Biofilms

Samples for the morphostructural analysis of bacterial aggregates were obtained from the experiments determining the degree of bacterial autoaggregation. The suspensions containing 1-h aggregates were transferred to Eppendorf tubes (Bionovo, Legnica, Poland) and centrifuged for 5 min at 8000 rpm (Gusto High-Speed Mini Centrifuge; Heathrow Scientific LLC, Vernon Hills, IL, USA). The supernatant was removed from the pellet and the obtained samples were then directed for analysis using SEM and TEM (see below for exact procedures). Regardless of the above experiments, the morphostructure of biofilm cells adhered to the solid surface was also determined. For this purpose, sterile 0.5 cm^3^ fragments of Columbia agar + 10% horse blood were inserted into each well of a 12-well microtiter plate (Bionovo, Legnica, Poland) containing 1 mL of BHI broth + 2% FCS and a bacterial suspension (10^8^ CFU/mL). Biofilm was obtained by means of adhesion of planktonic bacterial cells from the medium and establishment of the growth on this solid carrier [the so called “adsorption-incubation” method (Żywicka et al., 2020)]. The cultivation was carried out for one day and then the agar fragments with the immobilized bacteria were placed in Eppendorf tubes with 0.5 mL of a saline solution. Next, the agar fragments were centrifuged for 5 sec at 1500 rpm to remove loosely adhered bacterial cells. The agar fragments containing biofilms were then submitted for a SEM analysis.

Bacterial aggregates or agar fragments with attached biofilms, both contained in Eppendorf tubes, were flooded with 0.5 mL of a 2.5% solution of glutaraldehyde (Sigma-Aldrich, St. Louis, MO, USA) and incubated by a one day at 4°C. After the process of fixation, samples were centrifuged briefly and washed three times in 0.1 M cacodylate buffer (Sigma-Aldrich, St. Louis, MO, USA). Later, samples were passed through an increasing ethanol concentration gradient (30%, 50%, 70%, 90% and 99.8%). The obtained samples were sputtered with a carbon layer (EM ACE600, Leica Microsystems, Wetzlar, Germany) and observed with a Scanning Electron Microscope Auriga 60 (Oberkochen, Germany).

Apart from the observation using SEM, the analysis of the morphostructure of bacterial aggregates was also extended to TEM. Bacterial aggregates were fixed in a 2.5% solution of glutaraldehyde (Sigma-Aldrich, St. Louis, MO, USA) and centrifuged briefly (5 min). Contrasting of samples was performed in the dark using 2% uranyl acetate by 8 h and 2% osmium tetroxide by 2 h. The biological materials was then passed through an ascending alcohol series (30%, 50%, 70%, 90% and 99.8%). The samples prepared in this way were embedded in epoxy resin (Agar Low Viscosity Resin Kit, Agar Scientific Ltd., Stansted, UK). Sections of 60 nm were prepared from the resin blocks and placed on copper grids size 400 Mesh (Sigma-Aldrich, St. Louis, MO, USA) with formvar film and carbon coating. Imaging was performed using a JEOL JEM-1200 EX II microscope (Tokyo, Japan).

### 2.5 Statistical Analysis

The statistical significance of data within and between tested groups was determined by the Kruskal Wallis test with Bonferroni correction. Correlation analysis was performed with the Spearman correlation test. For all the tests, RStudio and a significance level of α = 0.05 were used.

## 3 Results

### 3.1 Primary Selection of Bacterial Strains

For the purpose this article, we selected 24 strains of *H. pylori* with different resistances to three antibiotics against which a disturbingly high level of resistance worldwide is observed now, i.e., CLR, MTZ and LEV (Savoldi et al., 2018). To investigate the relationship between biofilm production and different variations in antibiotic resistance profiles, we chose three representatives from each of the following groups: antibiotic-sensitive, mono-resistant (CLR, MTZ or LEV), double-resistant (CLR+MTZ, CLR+LEV and MTZ+LEV) and multidrug-resistant (CLR+MTZ+LEV) (**Supplementary Table 1**). All the strains included in this study came only from primary infected (non-eradicated) patients with gastritis, thus reducing the eventual impact of both repeated exposure of bacterial isolates to antibiotics targeting specifically this pathogen (secondary infections) and the modulatory effect of pathologically-altered environment of stomach (peptic ulcers and gastric cancer) on the phenotypic features of the strains (Soto-Girón et al., 2015; Ailloud et al., 2019).

### 3.2 Classification of Strains and Relationship Between Resistance and Biofilm Production

**Figure 1:**
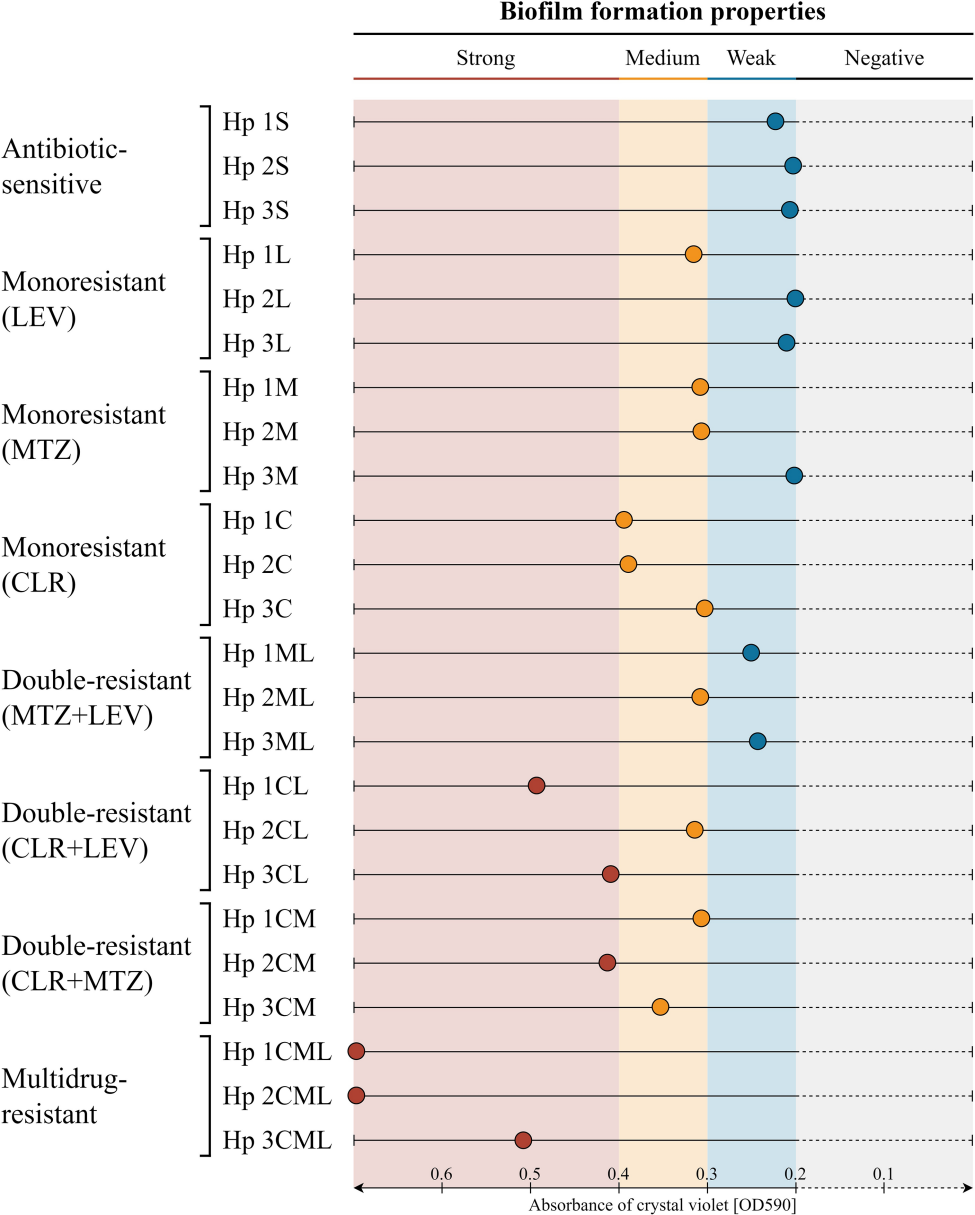
Graphical representation of the biofilm formation of 24 clinical *H. pylori* strains in static conditions and a 3-day incubation, determined using a crystal violet staining method. The *H. pylori* strains were classified as strong, medium and weak biofilm producers when the OD_590_ of the crystal violet solution was equal to ≥ 0.4, 0.4 - 0.3, 0.3 - 0.2, respectively. Values obtained for the pure medium, being a negative control, were ​​< 0.2 and indicated the lack of biofilm production by the tested strain. The tests were performed in three biological replications with six technical repetitions (n ​​= 18/strain). CLR, clarithromycin; MTZ, metronidazole; LEV, levofloxacin.

In the first stage of the research, we determined the ability of *H. pylori* strains to produce biofilm under static conditions with the use of a crystal violet staining method (**Figure 1**). Out the 24 *H. pylori* strains, 8 and 10 of them were categorized as weak and medium biofilm producers, respectively. The least numerous group were strong producers of this structure, constituting a quarter of the tested strains (6/24).

**Figure 2:**
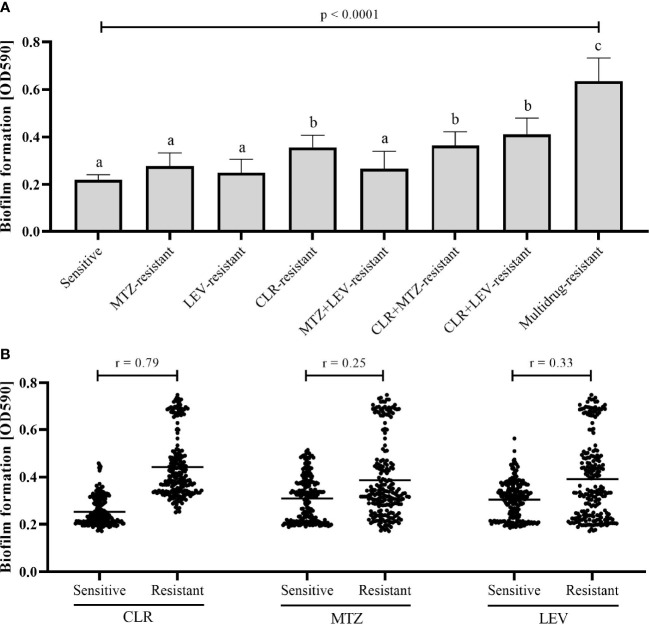
Correlation between the biofilm formation capacity of 24 clinical *H. pylori* strains and their resistance profile **(A)** or resistance to a specific type of antibiotic **(B)**. Determination of biofilm production was performed in static conditions and a 3-day incubation using a crystal violet staining method. The correlation between the biofilm formation and resistance profile **(A)** was calculated by the Kruskal Wallis test and columns with the same subscript letters (a, b, c) are not significantly different from each other (p > 0.05). The correlation between the biofilm formation and resistance to a specific type of antibiotic **(B)** was calculated by the Spearman correlation test, where 0 means no dependence and 1 a very strong dependence. The tests were performed in three biological replications with six technical repetitions (n ​​= 18/strain). CLR, clarithromycin; MTZ, metronidazole; LEV, levofloxacin.

We noticed significant differences in the biofilm production according to the antibiotic resistance profile of the strains (**Figure 2A**). The weakest biofilm producers were strains with antibiotic sensitivity, mono-resistance to MTZ or LEV, and double-resistance to both of these antibiotics (MTZ+LEV). The next group were strains with mono-resistance to CLR or having resistance to CLR and one additional antibiotic (CLR+MTZ or CLR+LEV), for which we observed a significantly higher biofilm formation potential compared to the first group (Kruskal-Wallis, p < 0.0001). The last group was represented by multidrug-resistant strains (CLR+MTZ+LEV), which produced the highest amount of biofilm (Kruskal-Wallis, p < 0.0001). From the results obtained above, we found existence of a strong correlation between the biofilm production and resistance to CLR (Spearman’s correlation r = 0.79), but not MTZ (Spearman’s correlation r = 0.25) or LEV (Spearman’s correlation r = 0.33) (**Figure 2B**). A detailed pairwise comparative analysis of biofilm formation properties of all the tested *H. pylori* strains is presented in **Supplementary Figure 1**.

Confirmation of the above observations was made by performing similar experiments for two reference strains: *H. pylori* Tx30a, representing antibiotic-sensitive strains being the weakest biofilm producers, and *H. pylori* ATCC 700684, a strain with mono-resistance to CLR, which based on this study was significantly correlated with the production of biofilm. The amount of biofilm formed by *H. pylori* ATCC 51932 and *H. pylori* ATCC 700684 corresponded to mean crystal violet absorbance values of 0.298 and 0.759, respectively. This classified them as a weak and strong biofilm producer, respectively, and supported independently our previous observations.

Based on the obtained results, we directed three the weakest (2S, 3M and 2L) and three the strongest (1CML, 2CML and 3CML) biofilm producers to the next stages of our experiments.

### 3.3 Validation of the Methodology Determining Biofilm Production Under Flow Conditions

To our knowledge, our research group is the first to use a microfluidic system in the comparative analysis of biofilm formation of *H. pylori* strains. For this reason, this step of experiments was associated with the methodology validation. In the first stage, we were faced with the task of selecting a functional model from the two classically used in studies of biofilm formation under microfluidic conditions (Gomes and Mergulhão, 2021): (1) a constant flow of medium containing bacteria and a measurement of the ability to adhere and form biofilm under a constant shear force pressure, or (2) a continuous flow of medium without bacteria and a measurement of the ability to grow biofilm only from pre-adhered cells/microaggregates. It turned out that only the first model provided suitable growth of the tested *H. pylori* strains (see further stages of the experiments), while for all the six tested *H. pylori* strains in the second model we did not observe any biomass increase (example *H. pylori* M172: **Supplementary Video 1**).

Knowing that only the first type of model ensured the growth of *H. pylori* strains, in the next stage of validation we determined two additional parameters of the experiments, i.e., the speed of medium flow (0.1 dyne/cm^2^, 0.15 dyne/cm^2^ or 0.2 dyne/cm^2^) and the incubation length (24 h, 48 h or 72 h). It turned out that while in the case of strong biofilm producers the development of biofilm was achieved using all three rates of the medium flow, in weak biofilm producers only the lowest speed ensured optimal growth of this structure (data not shown). As we were hoping to determine not only the amount of biofilm, but also measure some of its parameters (such as the composition of the biofilm matrix), in the current research we decided to choose a medium flow rate of 0.1 dyne/cm^2^. In the next step of validation, we decided to optimize the incubation length. In this case, it appeared that one day was the most appropriate in the model we adopted as the longer cultivation of strong biofilm producers contributed to disturbances in the experimental readings. It was directly related to the detachment of large biofilm fragments and their eventual collision with other biofilm fragments or microcolonies co-existing within microcapillaries. This situation was particularly well visible when observing strong biofilm producers, for which much faster development of the biofilm biomass was noticed (**Supplementary Videos 2** **–** **4**). Accordingly, the extended incubation periods of 48 h and 72 h were rejected.

In summary, in further steps of research the following model was used as the most optimal, i.e., formation of biofilm by *H. pylori* strains subjected to a constant flow of medium containing bacterial cells at a rate of 0.1 dyne/cm^2^ for 24 h.

### 3.4 Production of Biofilm Under Flow Conditions

**Figure 3:**
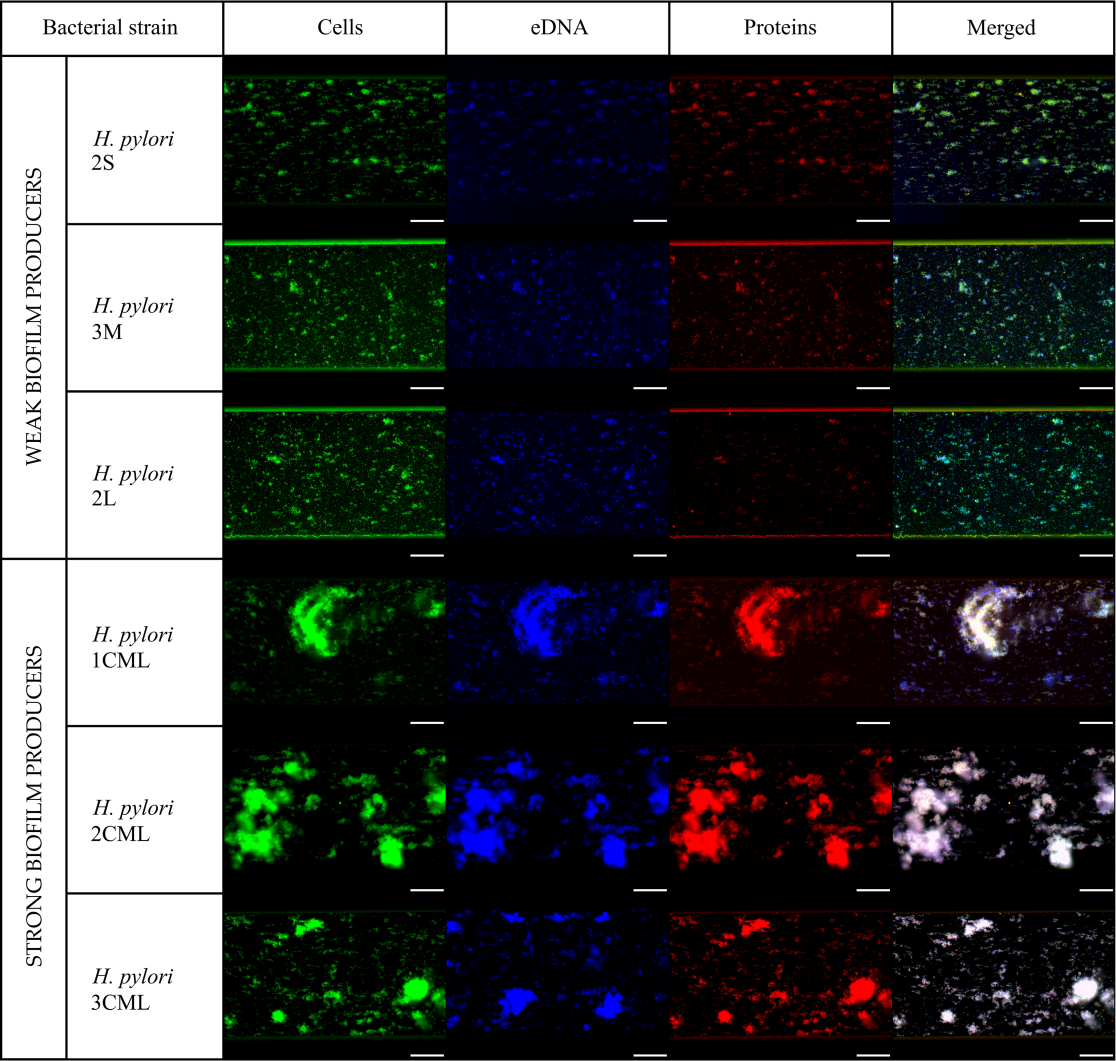
Representative fluorescence microscopy photographs of biofilms produced by six selected *H. pylori* strains incubated for 24 h under microfluidic conditions. Biofilms were stained by SYTO9 (green), DAPI (blue) and SYPRO RUBY (red) to enable determination of the bacterial biomass, extracellular DNA and extracellular proteins of the biofilm matrix, respectively. Scale bars show 20 µm.

**Figure 4:**
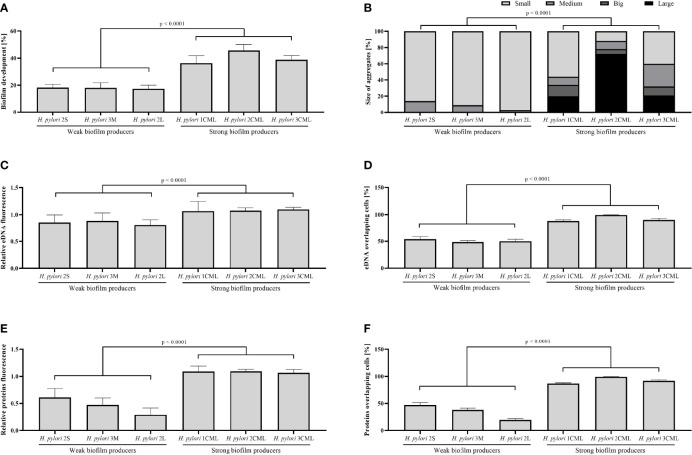
Numerical data obtained by the analysis of biofilms produced by six selected *H. pylori* incubated for 24 h under microfluidic conditions. The data include biofilm development **(A)**, size of aggregates with biofilm **(B)**, relative fluorescence of eDNA **(C)** and proteins **(E)**, and a percentage rate of eDNA **(D)** and proteins **(F)** overlapping bacterial cells in biofilms. The development of biofilm was determined based on the degree of microcapillary coverage. The fluorescence intensity of eDNA or proteins was normalized to fluorescence of the cellular biomass. The amount of eDNA or proteins overlapping bacterial cells was calculated based on the degree of co-localization of fluorescence signals and indicated the rate of bacterial coverage by the matrix components. The size of aggregates was normalized to dimensions of the microcapillary canal in the examined photo and clusters of bacterial cells corresponding to > 1%, 1% - 0.5%, 0.5% - 0.1% and < 0.1% of the microcapillary size were categorized as large, big, medium and small, respectively. The total area occupied by aggregates of a give category was calculated and presented as a percentage share in all aggregates formed. The microfluidic assays were performed in six biological replications with three technical repetitions constituting three different fragments of microcapillaries (n = 18/strain).

We observed that a one-day cultivation of *H. pylori* strains in microfluidic conditions contributed to the formation of biofilm of all the six tested strains, while with significant differences between both studied groups (strong vs. weak biofilm producers). Strong biofilm producers created more than twice as much biofilm, interpreted as the degree of microcapillary coverage, than weak producers of these structures (36.28 ± 5.73% to 45.66 ± 4.71% vs 17.38 ± 2.79% to 18.21 ± 2.49%; Kruskal-Wallis, p < 0.0001) (**Figures 3**, **4A**). The analysis of sizes of aggregates formed within microcapillaries showed the existence of a significant correlation between the dimensions of these structures and the ability to form biofilm (**Figures 4A, B**). This situation was particularly well visible when comparing the weakest biofilm producer – *H. pylori* 2L (2.7% and 97.3% of aggregates being medium- and small-sized, respectively) and the strongest biofilm producer – *H. pylori* 2CML (71.8%, 5.6%, 10.3% and 12.3% of aggregates classified as large, big, medium and small, respectively). It is also worth mentioning that for all three tested strains of weak biofilm producers the ability to form only medium and small aggregates was noticed (**Figure 4B**).

The next step in the analysis of the obtained results was associated with determining the degree of fluorescence of the most important components of the *H. pylori* biofilm, i.e., bacterial cells (green fluorescence), eDNA (blue fluorescence) and matrix proteins (red fluorescence). Again, we noticed the existence of large differences between the two groups of *H. pylori* strains. We observed statistically significant discrepancies in the intensity of eDNA fluorescence normalized to fluorescence of the cellular biomass between the studied groups, i.e., 1.07 ± 0.1 to 1.1 ± 0.04 vs 0.81 ± 0.07 to 0.89 ± 0.09 for strong and weak biofilm producers, respectively (Kruskal-Wallis, p < 0.0001; **Figure 4C**). We detected even more significant differences when analyzing the fluorescence of proteins normalized to fluorescence of the cellular biomass, being equal to 1.07 ± 0.06 to 1.1 ± 0.03 vs 0.29 ± 0.12 to 0.61 ± 0.16 for strong and weak biofilm producers, respectively (Kruskal-Wallis, p < 0.0001; **Figure 4E**). We also set out to determine the degree of co-localization of fluorescence signals as a parameter giving information about the coverage of bacterial cells by the tested matrix components. For both types of components the degree of eDNA/bacteria and proteins/bacteria co-localization was significantly higher for strong biofilm producers (88.07 ± 2.2% to 99.22 ± 0.4% and 86.72 ± 1.01% to 99.23 ± 0.48%, respectively) than for weak biofilm producers (48.35 ± 2.66% to 53.98 ± 4.64% and 19.54 ± 2.64% to 47.12 ± 4.66%, respectively) (Kruskal-Wallis, p < 0.0001; **Figures 4D, F**).

Summarizing the above part of the experiments, using the microfluidic conditions we were able to confirm the crystal violet-based assessment of biofilm production abilities of the tested *H. pylori* strains. Additionally, we showed that under conditions of the constant medium flow strong biofilm producers created well-developed biofilm structures composed largely of both eDNA and proteins.

### 3.5 Extension of Research on Autoaggregation and Characterization of Bacterial Aggregates

When culturing *H. pylori* strains under microfluidic conditions, we noticed some differences in the sizes of aggregates/clusters they formed (**Figure 3**). Therefore, in a later stage we decided to better characterize the above structures and the course of autoaggregation process.

**Figure 5:**
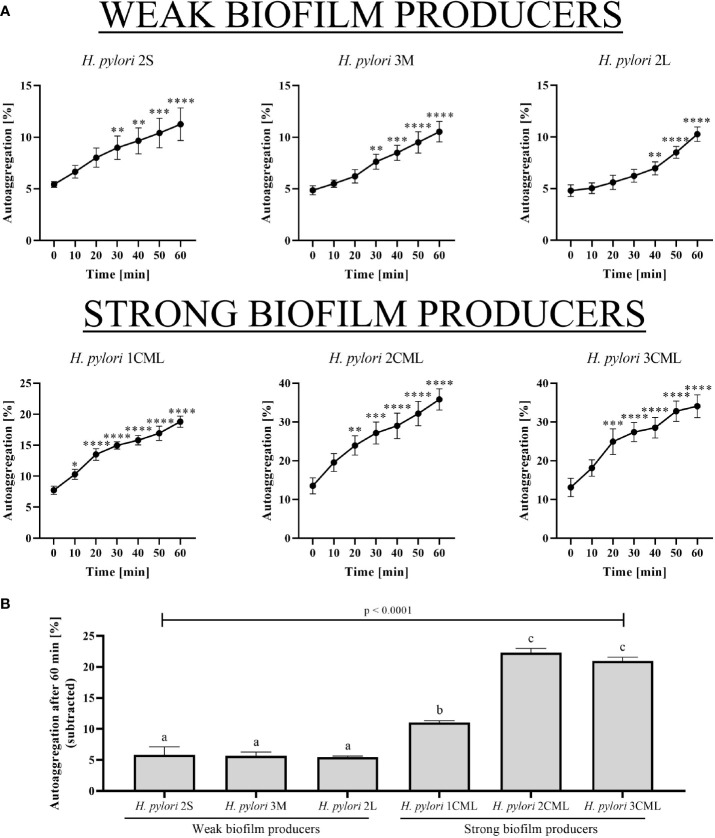
The time-dependent autoaggregation capacity of six selected *H. pylori* strains **(A)** and the final autoaggregation rate obtained after a 1-h incubation **(B)**. The analysis was performed by observing the rate of coverage of the microscopy observation field in the obtained photos. The autoaggregation rate after 1 h was calculated by subtracting the initial autoaggregation value in the sample (T_0_). In the figure **(A)**, p-values represented by *, **, ***, and **** are equal to < 0.05, < 0.01, < 0.001, and < 0.0001, respectively. In the figure **(B)**, columns with the same subscript letters (a, b, c) are not significantly different from each other (p > 0.05). The tests was performed in three biological replications (n ​​= 3/strain).

For this purpose, we analyzed time-lapse photos of all the six tested *H. pylori* strains, which were taken every minute for a period of 1 h (**Figure 5A**). We observed that all three strains representing weak biofilm producers had a similar degree of autoaggregation under static conditions ranging from 5.47 ± 0.7% to 5.82 ± 1.57% (**Figure 5B** and **Supplementary Videos 5** **–** **7**). The twice as high degree of autoaggregation was recorded for the *H. pylori* 1CML strain, which was equal to 11.05 ± 0.9% after 1 h (**Figure 5B** and **Supplementary Video 8**). The highest rate of autoaggregation was observed for the other two strains of strong biofilm producers - *H. pylori* 2CML and *H. pylori* 3CML (22.3 ± 2.74% and 20.96 ± 2.96%, respectively; **Figure 5B** and **Supplementary Videos 9** **,** **10**).

**Figure 6:**
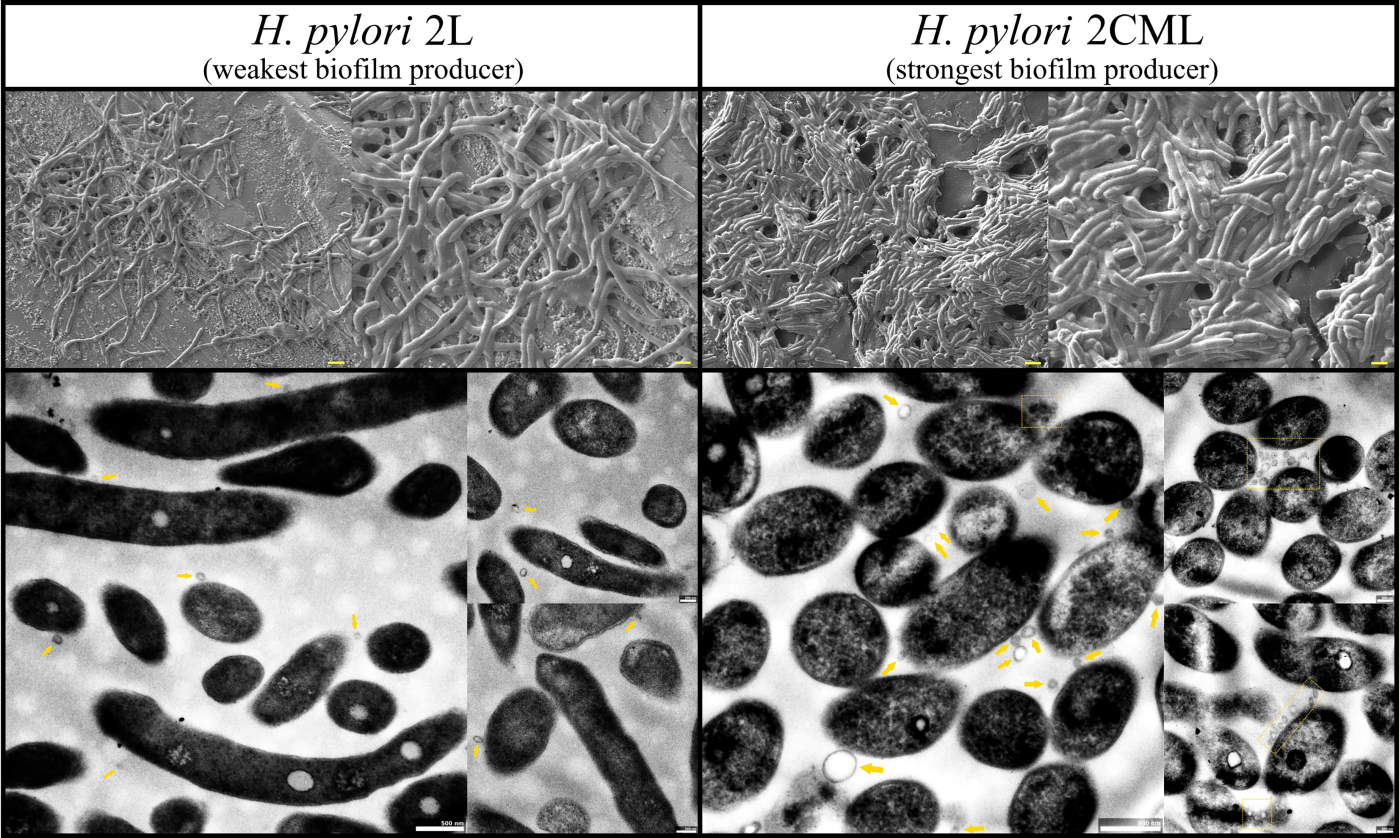
Representative SEM (top) and TEM (bottom) photographs of bacterial aggregates formed after a 1-h incubation of *H. pylori* 2L and *H. pylori* 2CML strains. The arrows point a single OMV, while the squares with a dashed line indicate a grouping of these structures. Scale bars for SEM show 2 µm and 1 µm for low- and high-magnification pictures, respectively.

An additional aspect of this part of the research was the morphostructural evaluation of 1-h biofilm-like aggregates. To obtain this, two strains with the contrary features were directed for this analysis, i.e., the weakest biofilm producer and at the same time the strain with the lowest tendency to autoaggregate - *H. pylori* 2L, and the strongest biofilm producer with the highest degree of autoaggregation - *H. pylori* 2CML. In the SEM images, the *H. pylori* 2L strain appeared mainly as long rods with occasionally co-existing cells of smaller sizes (short rods or coccoid forms) (**Figure 6**). In addition, it can be seen that bacteria are rather loosely organized and seen as a monolayer with numerous spaces between the bacterial cells. The TEM images showed additionally the presence of single, tiny (50-80 nm) outer membrane vesicles (OMVs) (**Figure 6**). The above-described characteristics differ significantly for the second tested strain. The SEM images of *H. pylori* 2CML demonstrated the predominance of short-sized cells (short rods or coccoid forms) (**Figure 6**). In contrast to the *H. pylori* 2L strain, the bacterial cells of *H. pylori* 2CML were characterized by the high compactness to each other and lower sharpness of the observed cells, probably as a result of matrix formation. Moreover, it can be perceived that the bacteria are organized into three-dimensional, multilayer structure perforated by concentric spaces, constituting most likely foci for the establishment of water channels. The TEM images of *H. pylori* 2CML showed again cells with shorter dimensions but also numerous OMVs with a large size range (50-300 nm, mainly about 100 nm) (**Figure 6**).

In conclusion, we observed large differences in the autoaggregation tendency between the strains representing strong and weak biofilm producers. Moreover, the morphostructural analysis of biofilm-like aggregates showed also discrepancies in the cellular morphology, the degree of cellular packing and spatial organization, as well as the amount of OMVs released into the environment.

### 3.6 Morphostructural Analysis of Biofilms

In the previous stages of our experiments, we observed that the tested *H. pylori* strains differ not only in the degree of autoaggregation and the morphostructure of aggregates produced in the initial stages of culture (**Figures 5**, **6**), but also in the amount of biofilm and dimensions of the clusters formed in the later stages of their development (**Figures 3**, **4**). For this reason, in the last stage of our research we decided to investigate the morphostructure of mature biofilms of *H. pylori*. For this purpose, we again selected two representatives with the most contrasting phenotype, i.e., *H. pylori* 2L and *H. pylori* 2CML.

**Figure 7:**
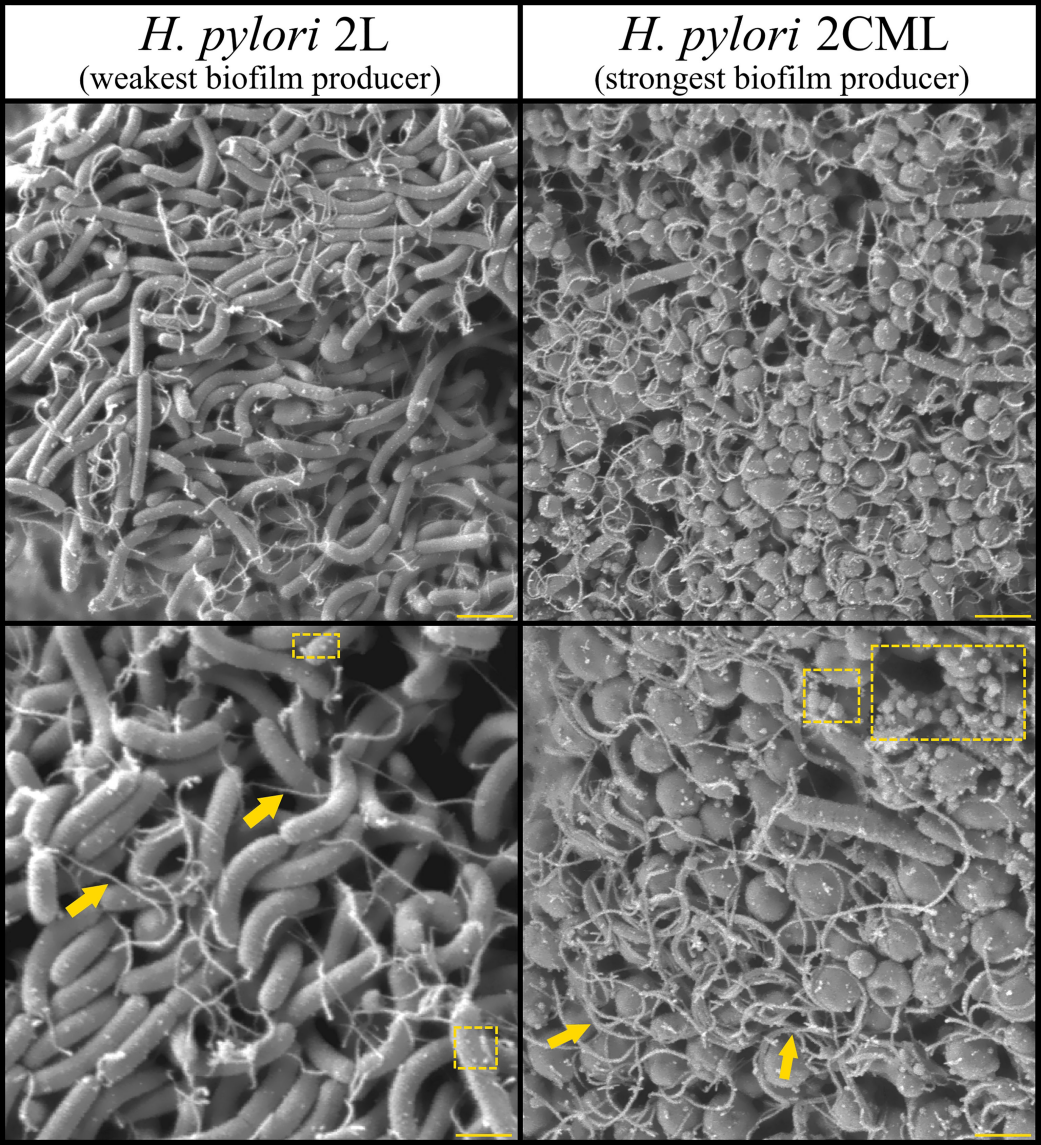
Representative SEM photographs of biofilms formed after a 24-h incubation of *H. pylori* 2L and *H. pylori* 2CML strains on the solid surface. The arrows mark flagella, while the squares with a dashed line indicate OMVs. Scale bars for the top images show 2 µm and for the bottom ones 1 µm.

The *H. pylori* 2L strain appeared mainly in a rod-shaped form with occasionally co-occurring coccoid forms. Bacterial cells were surrounded by a limited amount of tiny OMVs (20-80 nm) (**Figure 7**). Additionally, unlike for the electron microscopy images presenting bacterial aggregates, in this case we also noticed the presence of long, thin appendages extending from one pole of the cells and entwining cells in the close proximity. Based on the convergent observations and characteristics of these structures, we suspect that these are flagella (Hathroubi et al., 2018b; Hathroubi et al., 2020a). The images obtained for *H. pylori* 2CML again differed drastically from that acquired for the previous strain (**Figure 7**). Here, a coccoid form was the dominant morphotype. Apart from that, we observed the abundance of OMVs with relatively large size ranges (20-300 nm), as well as numerous flagella stabilizing the structure of biofilm.

Summing up, the analysis of the morphostructure of mature biofilms of a strong and weak producer of these structures confirmed the differences observed previously, including the morphology and the amount of OMVs. Very numerous flagella in the biofilm of a strong producer of this structure turned out to be a new aspect, unseen in electron microscopy images of bacterial aggregates.

## 4 Discussion

More than half of the people around the world are colonized by *H. pylori*, a bacterium associated with the development of serious gastric diseases contributing to the death of over a million people worldwide each year (Alexander et al., 2021). Unfortunately, the phenomenon of intensively spreading antibiotic resistance of *H. pylori*, in particular strains presenting multidrug-resistance, is a very serious threat to the effectiveness of therapies (Boyanova et al., 2019; Tshibangu-Kabamba and Yamaoka, 2021). The most extensively studied mechanisms of drug resistance of *H. pylori* include chromosomally encoded mutations in the drug target sites (Tshibangu-Kabamba and Yamaoka, 2021). Biofilm formation represent an independent mechanism that may contribute to the development and/or intensification of the antibiotic resistance, although the importance of this process in the above context is still poorly understood for *H. pylori* and undoubtedly requires further exploration (Yonezawa et al., 2015; Hathroubi et al., 2018a; Krzyżek et al., 2020). In response to this urgency, the aim of our article was to investigate the relationship between antibiotic resistance and biofilm formation of *H. pylori*.

When starting experiments screeningly determining the ability of *H. pylori* strains to produce biofilm, we made some methodological choices resulted from our review of the literature on this subject. Firstly, we used a liquid medium containing 2% FCS, a concentration of serum which according to Hathroubi et al. (2018b) was the most optimal to support the biofilm formation of *H. pylori*. In addition, nutrients depletion reduces the potential for rapid replication and thus limits the appearance of high biofilm formation values with the cellular biomass growth overpowering the biofilm matrix production (Zhang et al., 2014; Legner et al., 2019). Secondly, we applied a crystal violet staining method in the preliminary phase evaluating the ability of *H. pylori* strains to create biofilm. It is one of the most widely used techniques to classify bacteria in terms of their biofilm formation features (Azeredo et al., 2017), and the most commonly used in the study of *H. pylori* biofilms (Hathroubi et al., 2018a; Krzyżek et al., 2020). Therefore, in order to be able to refer our results to the data available in the literature, we chose this technique in the screening of the biofilm properties of *H. pylori* strains. In this context, we implemented the categorization developed by Fauzia et al. (2020), which was adopted to assess the biofilm capacities of *H. pylori*. However, to better reflect differences between the tested strains we introduced a slight modification to this classification, covering the addition of medium biofilm producers.

In the first stage of the research, using static conditions and the crystal violet staining method, we observed that resistance to CLR is positively correlated with biofilm production by *H. pylori* strains. The results showing this type of correlation are consistent with the observations of Fauzia et al. (2020), the only research apart from our current one, determining the relationship between antibiotic resistance of different *H. pylori* strains and their biofilm properties. We believe that our results have very important clinical implications as they confirm the importance of including CLR-resistant *H. pylori* strains within the WHO priority pathogen list (Tacconelli et al., 2018). It is also worth noting that in our research multidrug-resistant strains of *H. pylori* showed the highest degree of biofilm production, which seems to confirm the hypothesis about the interdependency of these two mechanisms as observed in other bacterial pathogens (Babapour et al., 2016; Devanga Ragupathi et al., 2020; Hosseini et al., 2020; Shadkam et al., 2021) and the urgent need to increase the amount of research on *H. pylori* biofilm as an important strategy preventing the spread of antibiotic resistance of this pathogen (Tshibangu-Kabamba and Yamaoka, 2021).

In the further stages of our research, we determined the ability of selected strongest and weakest biofilm producers of *H. pylori* to create these structures in microfluidic conditions. To our knowledge, our team is the first to adopt such a system in assessing biofilm formation of *H. pylori*. This system has already been used twice in our studies testing antibacterial activity of novel compounds against a selected *H. pylori* strain (Krzyżek et al., 2021; Spiegel et al., 2022), while the present article is the first in which microfluidic conditions have been used in the comparative evaluation of biofilm capacities of different *H. pylori* strains. For this reason, first steps of experiments were associated with the methodology validation. We found out that a measurement of the ability to grow biofilm only from pre-adhered cells/microaggregates was not suitable, because for all the six tested *H. pylori* strains we did not detect any biomass increase. We suspect that this was most likely related to the conversion of bacteria to viable but non-culturable (VBNC) state as a result of the lack of microaerophilic conditions during the pre-adhesion phase (a supply of gases is present only during the medium flow and is crucial for the proper growth of *H. pylori*) (Attaran and Falsafi, 2017; Hirukawa et al., 2018). We also noticed that incubation longer than one day was not appropriate in the chosen model as it stimulated the detachment of large biofilm fragments and their eventual collision with other biofilm fragments or microcolonies co-existing within microcapillaries. The situation described above is a natural phenomenon related to the maturation of biofilm exposed to shear forces, during which the biofilm integrity may be disrupted, leading to adhesive failure and detachment of this structure (Rumbaugh and Sauer, 2020; Guzmán-Soto et al., 2021). This process of biofilm sloughing, however, was associated with a very serious disturbance in the interpretation of our results, especially for strong biofilm producers, and hence the final incubation time was reduced to 24 h. After validating the methodology and adapting it to our model, we noticed significant differences between strong and weak biofilm producers. Under microfluidic conditions, strong biofilm producers created not only a significantly greater amount of biofilm, thus confirming the observations of screening experiments with crystal violet staining assays, but also showed a higher tendency of autoaggregation. This inspired us to extend the analysis of the tested *H. pylori* strains with the properties of their biofilm matrix and detailed imaging of their morphostructure.

In line with this, in the next step of our experiments were carried out the semi-quantitative fluorescence measurement of components within the biofilm created by *H. pylori*. In the analysis of the biofilm matrix composition we used fluorescence microscopy and dyes selectively labeling specific components, i.e., bacterial cells, eDNA and matrix proteins (Wilson et al., 2017; Hira et al., 2020). On the one hand, this type of choice was dictated by the results of Hathroubi et al. (2018b) and Windham et al. (2018), both of who using this technique showed that above components are the most important in the biofilm of *H. pylori*. On the other hand, this was imposed by the possibility of using up to three dyes simultaneously when assessing the fluorescent image by the naked eye (each subsequent one is a color derivative and prevents an exact visual reading of others in superimposed color images; the so called “color barrier”) (Pautke et al., 2005; Niwa et al., 2021). In line with the previous reports, we observed that both eDNA and proteins are important components of the *H. pylori* biofilm matrix. Importantly and innovatively, however, we noticed that strong biofilm producers created a significantly higher amount of eDNA and in particular proteins than weak producers of this structure. Moreover, the co-localization analysis revealed that most of the bacterial cells of strong biofilm producers, even those present in yet undeveloped aggregates, were covered by the matrix containing these components. This suggests a much faster production of eDNA and proteins constituting the matrix than this seen for weak biofilm producers. At this point, we would like to draw attention to our results showing that proteins constitute crucial components of the biofilm matrix of strong biofilm producers and that the capacity to create biofilms was correlated with resistance to CLR (an antibiotic inhibiting protein synthesis). By combining both these facts, we assume that the high efficiency of forming protein-dominated biofilms might be associated with great selection pressure for strong biofilm producers of *H. pylori* to create resistance to CLR. This hypothesis should be confirmed experimentally in the future, e.g., by a prolonged cultivation and multiple passages of antibiotic-sensitive *H. pylori* strains in the environment of sub-minimal inhibitory concentrations of CLR and determination whether changes in resistance to CLR are accompanied by an increase in biofilm formation capacities of the obtained isolates. The correlation between the resistance to antibiotics and the dominance of both proteins with eDNA in the biofilm matrix was observed also in strains of methicillin-resistant *Staphylococcus aureus* but not these with antibiotic sensitivity (Pozzi et al., 2012; McCarthy et al., 2015). A similar situation was noticed for another species of staphylococci - *Staphylococcus saprophyticus*, where biofilm matrix of a mixed chemical composition was observed in environmental strains (proteins, eDNA and polysaccharides), while proteinaceous biofilms dominated in clinical strains (Lawal et al., 2021). The above observations presenting the interplay between the biofilm matrix composition and the antibiotic resistance suggest that this phenomenon may be much more common and thus is worth further investigation. Although reports linking the composition of biofilm with antibiotic resistance in epidemiological terms are still sparse, increasing awareness is being paid to the role of DNABII proteins in the biofilm stabilization and biofilm-dependent generation of resistance to antibiotic pressure among different classes of microorganisms (Novotny et al., 2020; Goodman and Bakaletz, 2022; Rogers et al., 2022). Interestingly, in the review paper of Rogers et al. (2022) attention was drawn to the high conservativeness of the structure of these proteins among all analyzed bacteria qualified by WHO as priority ones, while the only representative for which data was missing was *H. pylori*. This information undoubtedly indicates a great need to conduct studies on the subject included in our research work as an important step aimed at increasing knowledge about biofilm forms of *H. pylori* and rising the degree of eradication of this pathogen.

The biofilm matrix is ​​a chemically and functionally diverse array of molecules collectively known as the “matrixome” (Karygianni et al., 2020). As proved hereby and discovered previously (Hathroubi et al., 2018b; Windham et al., 2018), the most important components of the biofilm matrix created by *H. pylori* are eDNA and proteins. eDNA is a polyanionic molecule that plays an important role in surface adhesion, horizontal genetic transfer, a chelation-dependent neutralization of antimicrobial peptides and some classes of antibiotics, and finally a stabilization of the biofilm structure (Campoccia et al., 2021; Panlilio and Rice, 2021). It is pointed out that the most important component of the biofilm matrix electrostatically reacting with eDNA are proteins (Campoccia et al., 2021). Protein components of biofilm include secretory extracellular proteins, adhesins and proteinaceous protrusions (e.g., flagella and pili), all of which are associated with adherence of microorganisms to the surface, autoaggregation and the development of biofilm spatial architecture (Fong and Yildiz, 2015). For *H. pylori*, it was discovered that OMVs can participate in the biofilm maturation through the presence of both eDNA and proteinaceous adhesins on their surface and lead to the formation of bridge interactions between bacterial cells (Yonezawa et al., 2009; Yonezawa et al., 2011; Grande et al., 2015; Yonezawa et al., 2017; Puca et al., 2019). Recent analyzes by Hathroubi et al., 2018b; Hathroubi et al., 2020a highlighted also flagella as an important proteinaceous component influencing the architecture of *H. pylori* biofilm. In line with the above observations, in the current research we saw a number of discrepancies in the morphostructure of both aggregates and biofilms formed by the strongest biofilm producer in comparison to these created by the weakest biofilm former. These differences included a greater degree of cellular packing, presence of shorter cells (short rods or coccoid forms) and a higher amount of OMVs and flagella entwining bacterial cells. In respect to other protein structures, a number of studies showed the key role of *H. pylori* efflux pumps in the development of biofilms (Attaran et al., 2017; Geng et al., 2017; Ge et al., 2018; Cai et al., 2020). In this regard, it is worth mentioning that strains of *H. pylori* presenting resistance to CLR (Geng et al., 2017) or multidrug-resistance (Ge et al., 2018; Cai et al., 2020) had a significantly higher level of efflux pumps’ expression compared to antibiotic-susceptible ones. These observations seem to complement our results showing significantly higher biofilm formation capacity in *H. pylori* strains with resistance to CLR or multidrug-resistance. In view of the above deliberations, we consider performing in the future an in-depth analysis of the relationship between antibiotic resistance of *H. pylori* strains and protein-eDNA interactions within the biofilm matrix as well as its proteome.

## 5 Conclusions

Biofilm formation is widely recognized as a critical mechanism of microorganisms to withstand antibiotic pressure. In line with this, we observed significantly higher biofilm formation capacity in *H. pylori* strains with resistance to CLR or multidrug-resistance. We also detected contrary phenotypical profiles when comparing the weakest and strongest biofilm producers. In that respect, strains with the most intensive biofilm-forming features created significantly more eDNA and in particular proteins within the biofilm matrix, had higher autoaggregation tendency and manifested morphostructural differences (a greater cellular packing, shorter cells and a higher amount of both OMVs and flagella). Furthermore, to the best of our knowledge, our research group was the first to adopt and prove usefulness of a microfluidic system in the comparative analysis of biofilm formation of *H. pylori* strains.

## Data Availability Statement

The original contributions presented in the study are included in the article/**Supplementary Material**. Further inquiries can be directed to the corresponding author.

## Author Contributions

PK designed the research. PK performed all the microbiological experiments. PM performed the statistical analysis and electron microscopy visualization. PK analyzed the data. PK prepared graphics. PK wrote an original draft of the manuscript. PK, PM, RG, and GG revised and edited the manuscript. PK supervised the work. All authors contributed to the article and approved the submitted version.

## Funding

This research was funded by Wroclaw Medical University, grant number SUBZ.A130.22.010. The Bioflux system was funded the National Centre for Research and Development (NCBiR) No. IA/SP/453975/2020.

## Conflict of Interest

The authors declare that the research was conducted in the absence of any commercial or financial relationships that could be construed as a potential conflict of interest.

## Publisher’s Note

All claims expressed in this article are solely those of the authors and do not necessarily represent those of their affiliated organizations, or those of the publisher, the editors and the reviewers. Any product that may be evaluated in this article, or claim that may be made by its manufacturer, is not guaranteed or endorsed by the publisher.
