# Supplementary material for: Biofilm Formation of Helicobacter pylori in Both Static and Microfluidic Conditions Is Associated With Resistance to Clarithromycin
Source: Front Cell Infect Microbiol. 2022 Mar 25;12:868905. doi: 10.3389/fcimb.2022.868905 (PMC8990135; doi:10.3389/fcimb.2022.868905)
Supplement: Supplementary file 1 [file DataSheet_1.docx]

Supplementary Materials


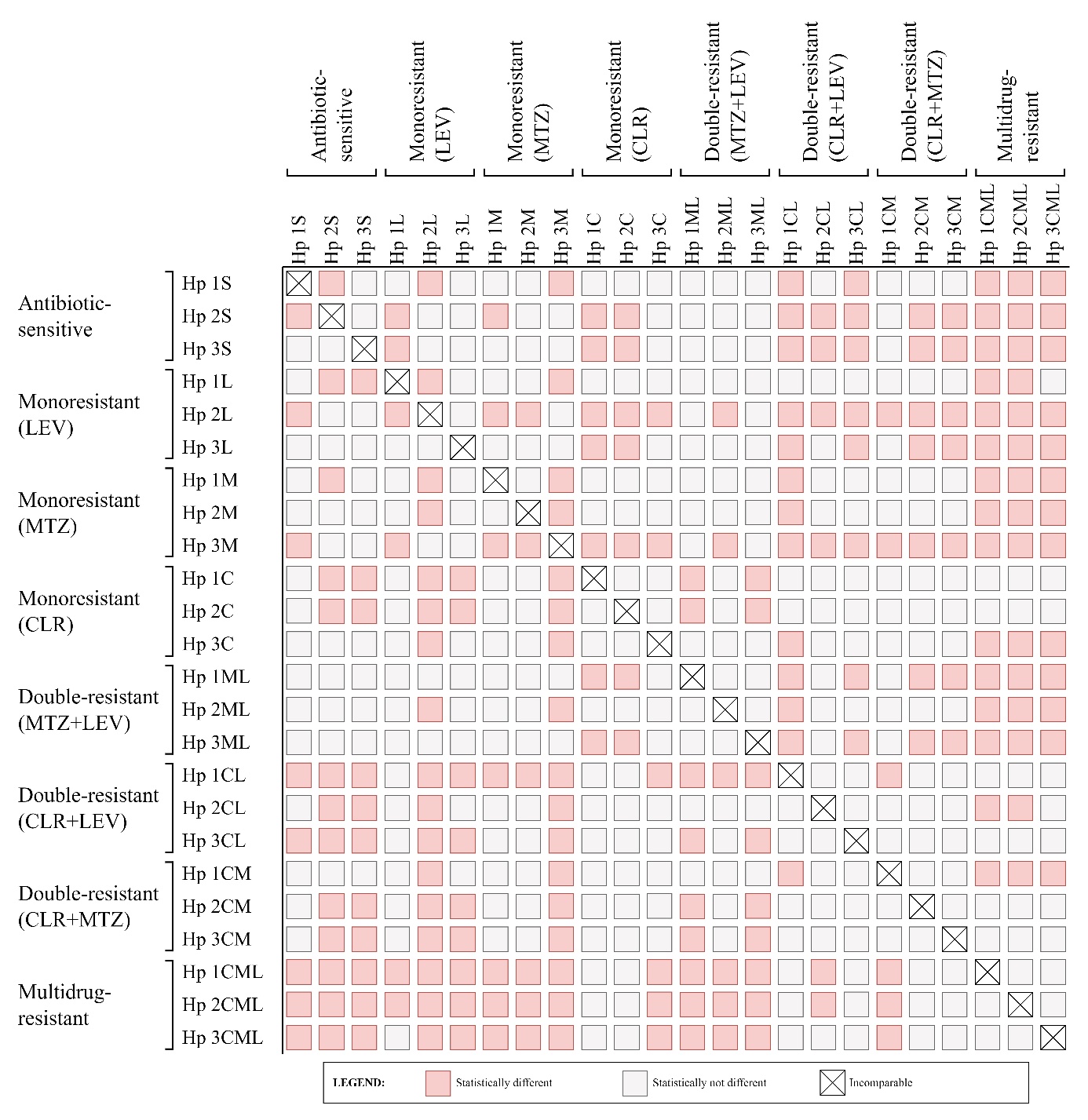


**Supplementary Figure 1.** A detailed pairwise comparative analysis of all tested *H. pylori* strains in term of their biofilm formation capacities in static conditions and a 3-day incubation. Determination of biofilm production was performed using a crystal violet staining method. Abbreviations: CLR, clarithromycin; MTZ, metronidazole; LEV, levofloxacin.

**Supplementary Table 1.** The minimum inhibitory concentrations ​​of antibiotics against the tested *H. pylori* strains.

| ***H. pylori* strains** | **Minimal inhibitory concentrations [µg/mL]** | | | | | **Antibiotic resistance profile** |
| --- | --- | --- | --- | --- | --- | --- |
|  | **CLR** | **MTZ** | **LEV** | **AMX** | **TET** |  |
| 1S | 0.016 (S) | 0.016 (S) | 0.032 (S) | 0.016 (S) | 0.125 (S) | Antibiotic-sensitive |
| 2S | 0.016 (S) | 0.25 (S) | 0.125 (S) | 0.016 (S) | 0.047 (S) |  |
| 3S | 0.023 (S) | 0.016 (S) | 0.002 (S) | 0.016 (S) | 0.016 (S) |  |
| 1C | 8  (R) | 0.125 (S) | 0.094 (S) | 0.016 (S) | 0.064 (S) | Mono-resistant to  CLR |
| 2C | 8  (R) | 0.047 (S) | 0.25 (S) | 0.032 (S) | 0.38 (S) |  |
| 3C | 3  (R) | 4 (S) | 0.25 (S) | 0.016 (S) | 0.125 (S) |  |
| 1M | 0.016 (S) | 256  (R) | 0.094 (S) | 0.016 (S) | 0.064 (S) | Mono-resistant to  MTZ |
| 2M | 0.016 (S) | 256  (R) | 0.016 (S) | 0.016 (S) | 0.094 (S) |  |
| 3M | 0.016 (S) | 256  (R) | 0.125 (S) | 0.016 (S) | 0.064 (S) |  |
| 1L | 0.064 (S) | 0.25 (S) | 3  (R) | 0.023 (S) | 0.032 (S) | Mono-resistant to  LEV |
| 2L | 0.016 (S) | 4 (S) | 32  (R) | 0.016 (S) | 0.047 (S) |  |
| 3L | 0.064 (S) | 0.25 (S) | 1.5  (R) | 0.016 (S) | 0.064 (S) |  |
| 1CM | 6  (R) | 256  (R) | 0.125 (S) | 0.016 (S) | 0.064 (S) | Double-resistant to  CLR and MTZ |
| 2CM | 256  (R) | 256  (R) | 0.094 (S) | 0.016 (S) | 0.094 (S) |  |
| 3CM | 6  (R) | 256  (R) | 0.38 (S) | 0.016 (S) | 0.064 (S) |  |
| 1CL | 64  (R) | 0.38 (S) | 1.5  (R) | 0.032 (S) | 0.094 (S) | Double-resistant to  CLR and LEV |
| 2CL | 12  (R) | 0.038 (S) | 3  (R) | 0.023 (S) | 0.032 (S) |  |
| 3CL | 3  (R) | 0.064 (S) | 1.5  (R) | 0.023 (S) | 0.125 (S) |  |
| 1ML | 0.016 (S) | 256  (R) | 6  (R) | 0.016 (S) | 0.094 (S) | Double-resistant to  MTZ and LEV |
| 2ML | 0.032 (S) | 256  (R) | 32  (R) | 0.023 (S) | 0.19 (S) |  |
| 3ML | 0.064 (S) | 256  (R) | 12  (R) | 0.032 (S) | 0.125 (S) |  |
| 1CML | 24  (R) | 256  (R) | 32  (R) | 0.023 (S) | 0.125 (S) | Multidrug-resistant |
| 2CML | 256  (R) | 256  (R) | 32  (R) | 0.016 (S) | 0.047 (S) |  |
| 3CML | 24  (R) | 256  (R) | 32  (R) | 0.023 (S) | 0.125 (S) |  |
| ATCC 51932 | 0.016 (S) | 0.064 (S) | 0.032 (S) | 0.016 (S) | 0.047 (S) | Antibiotic-sensitive |
| ATCC 700684 | 6  (R) | 0.064 (S) | 0.032 (S) | 0.016 (S) | 0.047 (S) | Mono-resistant to  CLR |

The determination of resistance of *H. pylori* strains was performed using E-tests. According to the EUCAST recommendations the resistance breakpoints were > 0.5 µg/mL, > 8 µg/mL, > 1 µg/mL, > 0.125 µg/mL and > 1 µg/mL for clarithromycin (CLR), metronidazole (MTZ), levofloxacin (LEV), amoxicillin (AMX) and tetracycline (TET), respectively (EUCAST, 2022). Abbreviations: R, resistant; S, sensitive.
